# Genome-wide association meta-analysis of coronary artery disease and periodontitis reveals a novel shared risk locus

**DOI:** 10.1038/s41598-018-31980-8

**Published:** 2018-09-12

**Authors:** Matthias Munz, Gesa M. Richter, Bruno G. Loos, Søren Jepsen, Kimon Divaris, Steven Offenbacher, Alexander Teumer, Birte Holtfreter, Thomas Kocher, Corinna Bruckmann, Yvonne Jockel-Schneider, Christian Graetz, Loreto Munoz, Anita Bhandari, Stephanie Tennstedt, Ingmar Staufenbiel, Nathalie van der Velde, André G. Uitterlinden, Lisette C. P. G. M. de Groot, Jürgen Wellmann, Klaus Berger, Bastian Krone, Per Hoffmann, Matthias Laudes, Wolfgang Lieb, Andre Franke, Henrik Dommisch, Jeanette Erdmann, Arne S. Schaefer

**Affiliations:** 1 Charité – University Medicine Berlin, corporate member of Freie Universität Berlin, Humboldt-Universität zu Berlin, and Berlin Institute of Health, Institute for Dental and Craniofacial Sciences, Department of Periodontology and Synoptic Dentistry, Berlin, Germany; 2 Institute for Cardiogenetics, University of Lübeck, 23562 Lübeck, Germany; 3 DZHK (German Research Centre for Cardiovascular Research), partner site Hamburg/Lübeck/Kiel, Lübeck, 23562 Germany; 4 Department of Periodontology and Oral Biochemistry, Academic Centre for Dentistry Amsterdam (ACTA), University of Amsterdam and Vrije Universiteit Amsterdam, Amsterdam, The Netherlands; 5 Department of Periodontology, Operative and Preventive Dentistry, University of Bonn, Bonn, Germany; 6 Department of Pediatric Dentistry, School of Dentistry, University of North Carolina at Chapel Hill, Chapel Hill, USA; 7 Department of Epidemiology, Gillings School of Global Public Health, University of North Carolina at Chapel Hill, Chapel Hill, USA; 8 Department of Periodontology, School of Dentistry, University of North Carolina at Chapel Hill, Chapel Hill, USA; 9 Institute for Community Medicine, University Medicine Greifswald, Greifswald, Germany; 10 Unit of Periodontology, Department of Restorative Dentistry, Periodontology, Endodontology, Preventive Dentistry and Pedodontics, Dental School, University Medicine Greifswald, Greifswald, Germany; 11 Department of Conservative Dentistry and Periodontology, Medical University Vienna, School of Dentistry, Vienna, Austria; 12 Department of Periodontology, Clinic of Preventive Dentistry and Periodontology, University Medical Center of the Julius-Maximilians-University, Würzburg, Germany; 13 Department of Conservative Dentistry, Unit of Periodontology, University Medical Center Schleswig-Holstein, Campus Kiel, Germany; 14 University Heart Center Luebeck, 23562 Lübeck, Germany; 15 Department of Conservative Dentistry, Periodontology and Preventive Dentistry, Hannover Medical School, Hannover, Germany; 16 Department of Internal Medicine, Erasmus Medical Center, Rotterdam, The Netherlands; 17 Department of Internal Medicine section of Geriatrics, Amsterdam Medical Center, Amsterdam, The Netherlands; 18 Wageningen University, Division of Human Nutrition, Wageningen, The Netherlands; 19 Institute of Epidemiology and Social Medicine, University Münster, Münster, Germany; 20 Institute of Medical Informatics, Biometry and Epidemiology, University Clinic Essen, Essen, Germany; 21 Institute of Human Genetics, University of Bonn, Bonn, Germany; 22 Human Genomics Research Group, Department of Biomedicine, University Hospital of Basel, Basel, Switzerland; 23 Department of Medicine 1, University of Kiel, Kiel, Germany; 24 Institute of Epidemiology, Christian-Albrechts-University, Kiel, Germany

## Abstract

Evidence for a shared genetic basis of association between coronary artery disease (CAD) and periodontitis (PD) exists. To explore the joint genetic basis, we performed a GWAS meta-analysis. In the discovery stage, we used a German aggressive periodontitis sample (AgP-Ger; 680 cases vs 3,973 controls) and the CARDIoGRAMplusC4D CAD meta-analysis dataset (60,801 cases vs 123,504 controls). Two SNPs at the known CAD risk loci *ADAMTS7* (rs11634042) and *VAMP8* (rs1561198) passed the pre-assigned selection criteria (P_AgP-Ger_ < 0.05; P_CAD_ < 5 × 10^−8^; concordant effect direction) and were replicated in an independent GWAS meta-analysis dataset of PD (4,415 cases vs 5,935 controls). SNP rs1561198 showed significant association (PD[Replication]: P = 0.008 OR = 1.09, 95% CI = [1.02–1.16]; PD [Discovery + Replication]: P = 0.0002, OR = 1.11, 95% CI = [1.05–1.17]). For the associated haplotype block, allele specific *cis*-effects on *VAMP8* expression were reported. Our data adds to the shared genetic basis of CAD and PD and indicate that the observed association of the two disease conditions cannot be solely explained by shared environmental risk factors. We conclude that the molecular pathway shared by CAD and PD involves VAMP8 function, which has a role in membrane vesicular trafficking, and is manipulated by pathogens to corrupt host immune defense.

## Introduction

Coronary artery disease (CAD) has a well-established genetic basis and a strong inflammatory component (reviewed in^1^). An association between CAD and the common oral inflammatory disease condition, periodontitis (PD), was reported in several clinical and observational studies (previously reviewed^2^). Because of the high prevalence of CAD and PD this association is potentially of public health importance. Recent evidence indicates that the observed association between CAD and PD is independent of smoking^2^ and obesity^3^. However, it could be explained in parts by other shared risk factors like diabetes, and age. In this context, the knowledge of shared genetic risk variants could substantially contribute to the understanding of the mechanisms that underlie the epidemiological associations.

Recently, we demonstrated shared associations of two CAD risk loci, *ANRIL*^4–7^ and *PLG*^8,9^, with aggressive periodontitis (AgP), a severe early-onset form of PD. In addition, we observed suggestive evidence for shared association of a rare genetic variant at *VAMP3*^10^, located at a chromosomal region that was earlier described to be associated with increased colonization of oral periodontal pathogens^11^. AgP, which has a global prevalence of 0.1%^12^, is characterized by a particular early age of disease onset (<35 years of age), which is why patients with AgP generally do not suffer from late-onset diseases like CAD or diabetes. By contrast, chronic periodontitis (CP), a widespread form of PD, has a prevalence of >9% in adults aged ≥30 years^13^ and is mainly observed in the elderly. AgP and CP have a similar histopathology and can be considered as parts of the same disease spectrum, which attribute to the effects of different combinations of genetic risk loci that determine the individual immune response. Because AgP cases have a high heritability as reflected by the early-age of disease onset and are not confounded by risk factors such as diabetes and age, AgP is particularly suitable to explore the shared genetic basis of CAD and PD^14^. The identified variants can be subsequently validated for the relevance in the more moderate form of CP.

In the current study, we performed a meta-analysis using data from the “Coronary Artery Disease Genome-wide Replication and Meta-analysis plus The Coronary Artery Disease” (CARDIoGRAMplusC4D) consortium and based on genome-wide association studies (GWAS) datasets for AgP and CP. We provide evidence for the shared association of variant rs1561198 with CAD and PD, which has reported *cis*-effects on the expression of the adjacent gene *VAMP8*.

## Materials and Methods

### Participating studies

The discovery sample consisted of a GWAS of German AgP cases and controls, which was previously described^15^, and of a case-control GWAS meta-analysis of CAD from the CARDIoGRAMplusC4D consortium^16^. The replication was carried out with GWA studies of case-control samples of Dutch AgP^15^ and CP case-control samples of German^17^ and of European American descent^18^. Summary statistics of all studies were based on the additive model.

The German AgP sample (AgP-Ger) included 680 cases and 3,973 controls. Cases were recruited across Germany by the biobank Popgen^19^, University-Hospital Schleswig-Holstein, Germany. Controls originate from North- and West-Germany and were recruited from the Competence Network “FoCus - Food Chain Plus”^20^, the Dortmunder Gesundheitsstudie – DOGS^21^ and the Heinz Nixdorf Recall Studies 1–3^22^. Genotyping of the cases was performed on Illumina Omni Bead Chips and the imputation was based on the 1000 Genomes Phase 3 reference panel^23^.

The dataset of the CARDIoGRAMplusC4D consortium included an assembly of 60,801 cases and 123,504 controls from 48 studies, mainly of European, South Asian East Asian descent^16^. Genotyping was carried out using Affymetrix or Illumina platforms. Subsequent genotype imputation was based on the 1000 Genomes phase 1 version 3 reference. The meta-analysis was performed by using either the fixed-effects model or the random-effects model, depending on the level of statistical heterogeneity.

The Dutch AgP sample (AgP-NL) consisted of 171 cases of Dutch descent that were recruited from the ACTA (Academisch Centrum Tandheelkunde Amsterdam) and of 2,607 population representative controls from the B-Proof Study^24^, which were collected at Rotterdam and Wageningen. Genotyping of the cases and imputation was performed together with AgP-Ger.

The German CP (CP-Ger) sample consisted of 993 cases and 1,419 controls from two independent cross-sectional studies SHIP and SHIP-TREND, recruited at the University Medicine Greifswald^25–27^. Cases and controls were defined by contrasting subjects within the first vs the third tertile of proportion of proximal sites with attachment loss (AL) ≥4 mm. To increase the statistical power by enriching the case sample with early-onset phenotypes, subjects aged >60 years were excluded. Cases and controls were genotyped either with the Affymetrix Genome-Wide Human SNP Array 6.0 or the Illumina Human Omni 2.5 array and imputed on the 1000 Genomes Phase 1 reference.

The European American CP (CP-EA) sample included 958 severe (sev) CP cases, 2,293 moderate (mod) CP cases and 1,909 controls from the Atherosclerosis Risk in Communities (ARIC) Study^28^. Genotyping was carried out using the Affymetrix Genome-Wide Human SNP Array 6.0 and the subsequent genotype imputation was performed on the HapMap Phase II reference with individuals of Northern and Western European (CEU) ancestry.

### Discovery

In the discovery stage, we compared variants in GWAS of AgP-Ger and CAD regarding their effect direction and significance level. At first, variants with a minor allele frequency (MAF) <0.05 were filtered out and only variants with genotypes available for both study samples were kept.

Then we selected variants showing the same effect direction in AgP-Ger and CAD and surpassing the pre-assigned restrictions P_AgP-GER_ < 0.05 and P_CAD_ < 5 × 10^−8^. We chose these P-value thresholds to account for the huge difference in sample size (CAD sample ~35x larger than AgP-Ger sample). Subsequently, we pruned the remaining variants using intermediate linkage disequilibrium. We chose a pruning threshold of *r*^2^ ≥ 0.2 to account for association tails of variants arising from their low correlation with the truly associated haplotype block.

### Replication meta-analysis

Variants that passed the discovery step were taken forward to replication stage and meta-analysed in additional PD GWAS samples AgP-NL, CP-Ger, CP-EA-mod and CP-EA-sev. By default, we applied the fixed effects model. However, for variants having a high amount of heterogeneity, i.e. where the P-value of Cochran’s Q P(Q) <0.05 and heterogeneity index I^2^ > 0.5, we applied the random effects model instead. Correction for multiple testing was performed using the method of Bonferroni. Additionally, we used the adjustment method for shared controls of Zaykin and Kozbur to check the P-value inflation when combining CP-EA-mod and CP-EA-sev^29^.

### Statistical power calculation for the discovery stage

The statistical power of the AgP-Ger sample at a significance level of 0.05 was calculated with Genetic Association Study (GAS) Power Calculator^30^. When assuming an additive model and a prevalence rate of 0.1% for AgP in the general population^12^, risk variants with minor allele frequencies of >0.4 can be detected at a probability (power) of 0.8 if the genotype relative risk (GRR) is ~1.18. The GRR is an estimator for the OR.

### Functional annotation

Variants were annotated using the Genehopper database (DB)^31^. Genehopper DB integrates data of many public sources by applying periodically executed extraction, transformation and loading (ETL) processes. Specifically, we used integrated datasets of linkage disequilibrium (LD), expression quantitative trait loci (eQTL) mappings, topologically associated domain boundaries (TADs) and GWAS studies to annotate our findings.

Identified loci were annotated using LD information (correlation measures r^2^ and D’) from the European reference population (EUR) of 1000 Genomes Phase 3^23^. EQTL mapping information was gathered from Genotype-Tissue Expression project (GTEx)^32^, Haploreg v4.1^33,34^, GRASP v2.0^35^, GEUVADIS^36^, SCAN^37^, seeQTL^38^ and Blood eQTL Browser^39^. Information about TAD boundaries was taken from Dixon *et al*.^40^. TADs are genomic regions defined by mainly cell-type independent interactome boundaries representing a spatial compartment in the genome. Physical interactions of regulatory DNA elements and gene promoters occur more frequently within a TAD. Thus, we refer to *cis* regulation, if a SNP with a putative effect on gene expression (expression SNP; eSNP) resides upstream or downstream from the gene but within the same TAD. In contrast, if a gene is located in a different TAD than the corresponding eSNP, we define this as a putative trans-regulatory effect, being most likely affected indirectly, e.g. via intermediate genes in a pathway. This dataset contained TADs with a length of ~853 kilo base pairs (kb) in average (maximum length = 4.44 mega base pairs [mb], minimum [min] length = 0.8 kb). Variant consequence information was taken from Ensembl Variation DB and additionally, we annotated variants using combined annotation dependant depletion (CADD) score^41,42^. The CADD score is calculated by applying the formula −10 × log_10_ (rank/total) on each variant in a ranking of single nucleotide variants which is created by combining several other variant annotation scores using machine learning techniques. Accordingly, a CADD score of ≥5.22 indicates that the variant belongs to the 20% most deleterious substitutions in the human genome and a score ≥10 indicates that the variant belongs to the 10% most deleterious substitutions. To elucidate the relationship to other traits and diseases, we extracted information about phenotype associations from the NHGRI-EBI Catalog^43^.

### Genetic risk score calculation

In an additional analysis, we assessed the genetic relation of CAD and PD by calculating a genetic risk score (GRS) based on known CAD risk variants and their corresponding effect sizes in CAD and by applying this score to the sample of AgP-Ger and respective controls (Supplementary Table 1). The GRS was calculated per individual using the formula *GRS* = Σ*w*_*x*_*n*_*x*_, with *w*_*x*_ being the effect size (Odds ratio [OR]) and *n*_*x*_ being the number of risk alleles of variant *x*. GRS of cases and controls of AgP-Ger were then compared using basic statistics.

## Results

### Discovery meta-analysis

The variant sets of AgP-Ger and CAD consisted of 6,416,838 and 9,455,779 variants. A total of 5,519,261 genetic variants were existent in both studies. In the discovery stage which included AgP-Ger and CAD, 276 variants in three distinct loci at 9p21.3, 15q25.1 and 2p11.2 surpassed our pre-assigned selection criteria (Supplementary Table 1).

**Figure 1:**
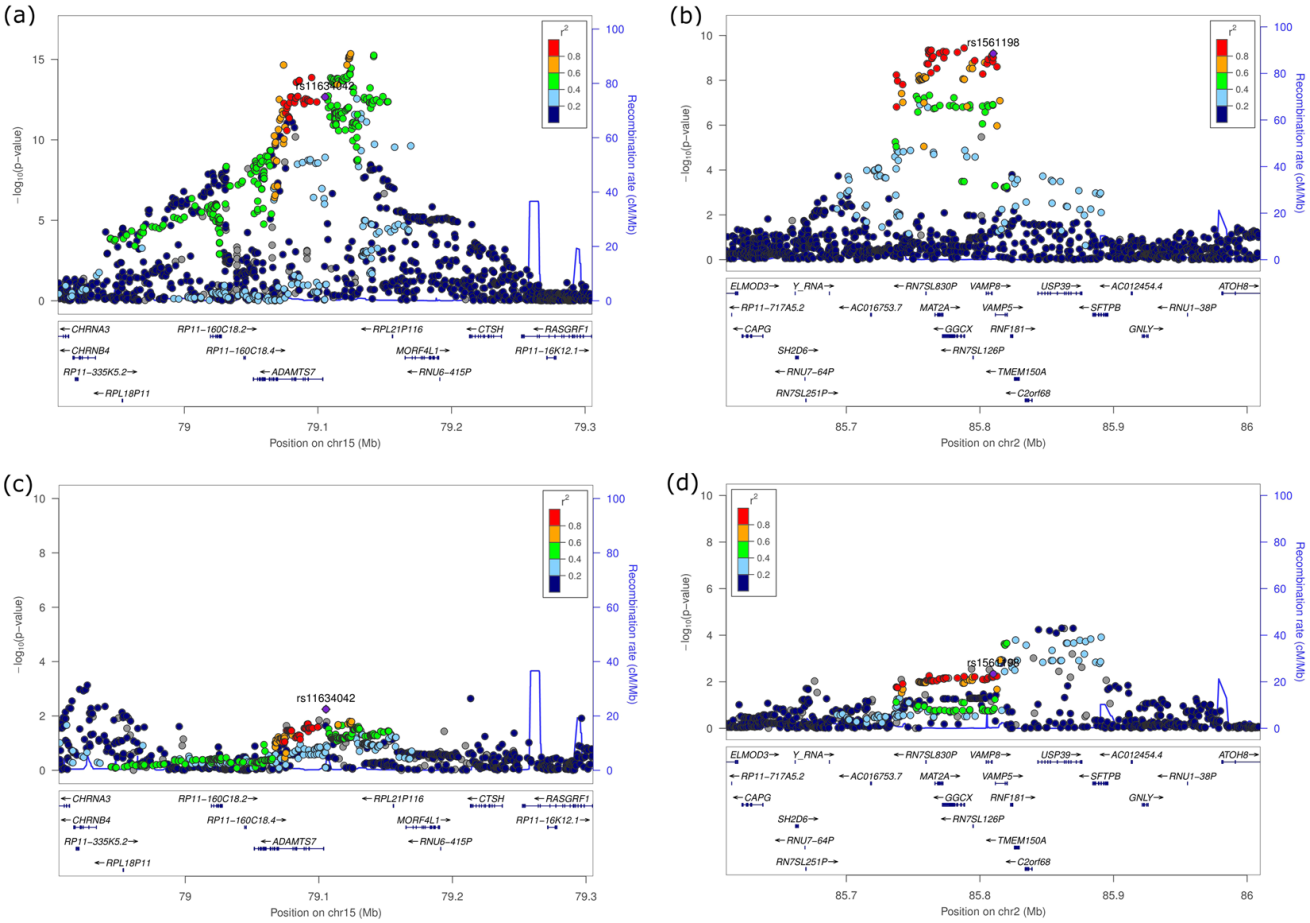
Regional association plots of the two loci at 15q25.1 and 2p11.2 that were identified in the discovery meta-analysis and not yet known to be associated with PD. The plots show a region +/−200 kb of the discovery lead variants. (**a**) rs11634042 in CAD (**b**) rs1561198 in CAD (**c**) rs11634042 in AgP-Ger and (**d**) rs1561198 in AgP-Ger.

Locus 9p21.3 reached the highest level of significance in PD with P = 1.23 × 10^−4^ (OR = 1.26, 95% CI = [1.12–1.41]) and P = 1.56 × 10^−32^ (OR = 1.21, 95% CI = [1.17–1.25]) for lead variant rs10116277 in AgP and CAD, respectively (Table 1 and Fig. 1). This single nucleotide polymorphism (SNP) is located in the intronic region of the large non-coding antisense RNA *CDKN*2*B-AS1* (alternative name *ANRIL*) which has been reported as risk factor for both AgP and CAD before^7,44^.

**Table 1:** Three loci were identified in the discovery step (AgP-Ger, CAD): 9p21.3, 15q25.1 and 2p11.2.

	Lead Variant	Locus	Nearest Gene(s)	A1	A2	EAF Cas	EAF Con	Stage	OR [95% CI]	P
1	rs10116277	9p21.3	CDKN2B-AS1	T	G	0.52	0.46	AgP-Ger	1.26 [1.12–1.41]	1.23E-04
								CAD	1.21 [1.17–1.25]	1.56E-32
2	rs11634042	15q25.1	MORF4L1	C	T	0.60	0.56	AgP-Ger	1.18 [1.05–1.32]	5.67E-03
								CAD	1.08 [1.06–1.10]	2.17E-13
								AgP-NL	1.00 [0.80–1.25]	1
								CP-EA-mod	1.00 [0.90–1.10]	9.53E-01
								CP-EA-sev	1.04 [0.91–1.20]	5.34E-01
								CP-Ger	0.96 [0.85–1.09]	5.40E-01
								**Pooled (Replication)**	**1.00 [0.94–1.06]**	**9.50E-01**
								**Pooled (PD)**	**1.04 [0.98–1.10]**	**1.99E-01**
3	rs1561198	2p11.2	VAMP8 - VAMP5	T	C	0.48	0.44	AgP-Ger	1.19 [1.05–1.34]	4.57E-03
								CAD	1.06 [1.04–1.08]	6.37E-10
						0.45	0.47	AgP-NL	0.96 [0.77–1.20]	7.10E-01
								CP-EA-mod	1.09 [1.00–1.20]	5.81E-02
								CP-EA-sev	1.09 [0.95–1.24]	2.08E-01
						0.46	0.43	CP-Ger	1.13 [1.00–1.28]	5.86E-02
								**Pooled (Replication)**	**1.09 [1.02–1.16]**	**6.79E-03**
								**Pooled (PD)**	**1.11 [1.05–1.17]**	**2.02E-04**

Since the locus at 9p21.3 is already a known shared risk locus for PD and CAD, we only took the loci at 15q25.1 and 2p11.2 forward to replication in PD (Pooled[Replication] = AgP-NL + CP-EA-mod + CP-EA-sev + CP-Ger). In the replication step, only locus 2p11.2 could be successfully replicated. At this locus, SNP rs1561198 showed the strongest association with PD in the overall PD sample (Pooled[PD] = AgP-Ger + AgP-NL + CP-EA-mod + CP-EA-sev + CP-Ger).

A1 = Effect allele; A2 = Non-effect allele; EAF = Effect allele frequency; Cas = Cases; Con = Controls; OR = Odds ratio; CI = Confidence interval; P = P-value; CAD = Coronary artery disease; AgP = Aggressive periodontitis; CP = Chronic periodontitis; Ger = German; NL = Dutch; EA = European American; sev = severe; mod = moderate.

The lead variant of the second locus at 15q25.1 was SNP rs11634042, intronic of the gene *MORF4L1* (Mortality factor 4 like 1) and 21 kb upstream of *ADAMTS7* (ADAM metallopeptidase with thrombospondin type 1 motif, 7) with P = 5.67 × 10^−3^ (OR = 1.18, 95% CI = [1.05–1.32]) and P = 2.17 × 10^−13^ (OR = 1.08, 95% CI = [1.06–1.10]) for AgP and CAD.

The third associated region at 2p11.2 showed association with P = 4.57 × 10^−3^ (OR = 1.19, 95% CI = [1.05–1.34]) and P = 6.37 × 10^−10^ (OR = 1.06, 95% CI = [1.04–1.08]) for variant rs1561198 in AgP-Ger and CAD, respectively. Variant rs1561198 is an intergenic SNP located 0.8 kb downstream of *VAMP8* (vesicle-associated membrane protein 8) and 1.5 kb upstream of *VAMP5* (vesicle-associated membrane protein 5). Also the loci at *MORF4L1* and *VAMP8* are already known susceptibility loci for CAD^16,45^, however they have not yet been reported to be associated with PD.

### Replication

**Figure 2:**
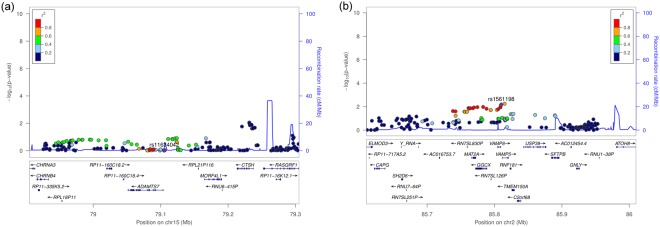
Regional association plots of (**a**) rs11634042 +/−200 kb at 15q25.1 and (**b**) rs1561198 +/−200 kb at 2p11.2 based on the pooled replication samples AgP-NL, CP-EA-mod, CP-EA-sev, CP-Ger.

Of the three loci that we identified in the discovery meta-analysis, *ANRIL* had repeatedly been replicated as a genetic risk factor of PD^7,46–48^. Accordingly, we only selected the two novel suggestive risk loci at 15q25.1 and 2p11.2 for replication. To address putative multiple independent association signals in these two loci, we first applied variant pruning on the 75 variants that passed our selection criteria in the discovery meta-analyses, using intermediate linkage disequilibrium of *r*^2^ ≥ 0.2 (Supplementary Table 2). Only the two lead variants rs11634042 at 15q25.1 and rs1561198 at 2p11.2 remained after pruning, indicating an association of a single haplotype block at each locus. Therefore, only SNPs rs11634042 and rs1561198 as well as their high LD variants (*r*^*2*^ ≥ 0.8; in the following we call these variant sets LD blocks) were selected for replication in PD (Supplementary Table 3). At 15q25.1, SNP rs11634042 (intronic of *MORF4L1*) had the lowest P-value in the replication meta-analysis, with P = 0.95 (OR = 1, 95% CI = [0.94–1.06]), showing no association with PD (Table 1 and Fig. 2).

At 2p11.2, SNP rs1561198 showed the strongest association (P = 0.007; OR = 1.09; 95% CI = [1.02–1.16]) among 15 out of a total of 38 strongly linked variants of this haplotype block, for which genotype data were available for all PD samples. The association of rs1561198 with PD was significant in the replication after correction for multiple testing, and after pooling all PD samples, SNP rs1561198 had a P-value of P = 2.02 × 10^−4^ (OR = 1.11; 95% CI = [1.05–1.17]).

A comparison of pooled P-value for CP-EA-mod and CP-EA-sev with and without adjustment for shared controls indicated a reasonable inflation of less than one potency (Supplementary Table 4).

### *In-silico* characterization of selected variant effects

Next, we investigated the associated genetic region at rs1561198 for putative regulatory effects on other genes and associations with other traits. First, we grouped genes in *cis* and -*trans* with respect to SNP rs1561198 and its 38 variants in the LD block using information about topologically associated domain boundaries (TADs). The LD block of rs1561198 is located within a TAD spanning 2 mega base pairs (mb) on chromosome 2. This TAD harbours 14 genes of which seven are protein coding (Supplementary Table 5). Examination of the 39 variants in the haplotype block of rs1561198 indicated study-wide significant *cis*- and *trans*-regulatory effects on multiple genes (Supplementary Table 6). Table 2 shows those study-wide significant eSNP effects in *cis* with P < 10^−10^ for blood and gastrointestinal tissues that, to our knowledge, have been reported to date. For these *cis*-located genes the strongest eQTLs were found in blood for *VAMP8* (Vesicle associated membrane protein 8; P = 9.8 × 10^−198^), *MAT2A* (Methionine adenosyltransferase 2A; P = 1.4 × 10^−76^), USP39 (P = 9.8 × 10^−38^), *VAMP5* (P = 8.2 × 10^−32^) and *GGCX* (Gamma-glutamyl carboxylase; P = 3.7 × 10^−33^).

**Table 2:** *Cis* eQTLs with P < 10^−10^ in blood and/or gastrointestinal tissue for the LD block of replicated SNP rs1561198.

Lead SNP	Gene	Samples (Best P-value)
rs1561198	GGCX	Whole blood (3.7e-33), Artery - Tibial (6.2e-25), Artery - Aorta (1.2e-21), Esophagus - Mucosa (2e-19), Atherosclerotic aortic root (1.8e-15), Cells - EBV-transformed lymphocytes (1.8e-14), Muscle skeletal (6.3e-13), Heart - Left ventricle (5.5e-11)
rs1561198	MAT2A	Blood (1.4e-76), Artery - Mammary (1.4e-14)
rs1561198	USP39	Peripheral blood (9.8e-38)
rs1561198	VAMP5	Peripheral blood (8.2e-32), Blood (1.5e-11), Muscle skeletal (1.5e-11)
rs1561198	VAMP8	Peripheral blood (9.8e-198), Blood (1.4e-76), Esophagus - Mucosa (2.9e-35), Cells - Macrophages (1.4e-24), Cells - Monocytes (1.6e-19)

The smallest P-value per sample is given in brackets.

**Figure 3:**
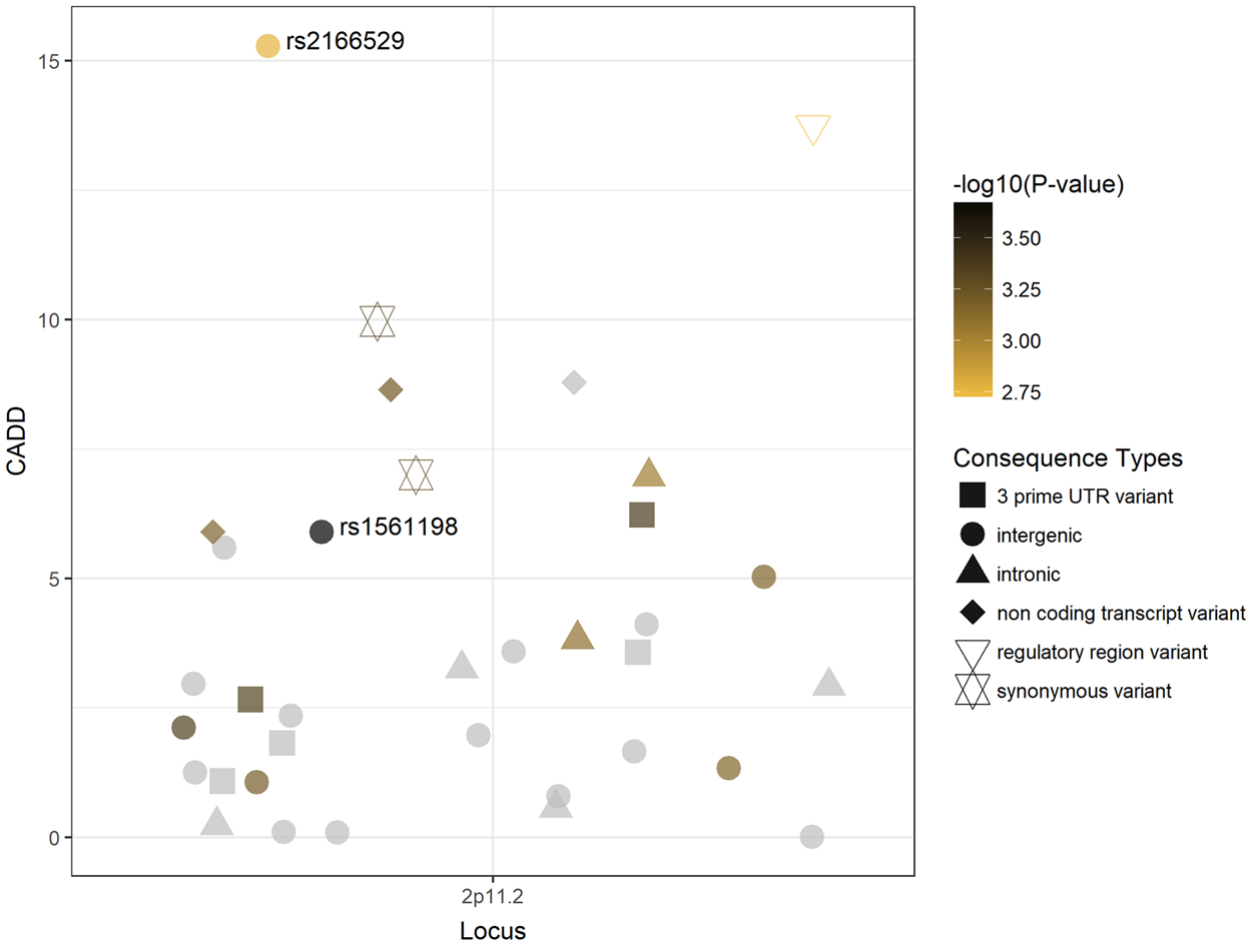
rs1561198 and the variants in its LD block (*r*^2^ ≥ 0.8) at 2p11.2 are plotted against their CADD scores. The colour of each symbol refers the pooled P-value of all PD samples. Within the LD block, rs1561198 showed the lowest association P-value whereas rs2166529 showed the second highest association P-value and the highest CADD score. Since CADD scores are calculated for SNPs only, we four didn’t include four insertions and deletions in this plot.

Additionally, we assessed the relative pathogenicity of the variants in the haplotype block using the combined annotation dependant depletion score (CADD) to explore their potential of being causal for the observed association (Supplementary Table 7). Lead SNP rs1561198 was among 12 variants with CADD ≥5, indicating a comparably high probability for having a causal effect (Fig. 3). The intergenic SNP rs2166529 (r^2^ = 0.82 with rs1561198), was assigned with the highest CADD score (15.29). Spearman correlation of *r*_*s*_ = 0.26 (P = 0.34; Alternative hypothesis: true r_s_ is not equal to 0) between CADD scores and P-values of the associations with PD indicated a low non-significant correlation.

Furthermore, we searched the LD block of rs1561198 for the presence of GWAS lead SNPs of other diseases and traits as listed in the in the NHGRI-EBI GWAS Catalog with P < 10^−5^. According to the catalogue, the LD block is associated with CAD (rs7568458, P = 4 × 10^−10^), myocardial infarction (SNP rs10176176, P = 3 × 10^−10^) and prostate cancer (rs3731827, P = 3 × 10^−9^) with genome-wide significance (Supplementary Table 8). Moreover, the chromosomal region at rs1561198 +/− 500 kb is associated with additional phenotypes including genome-wide significant associations with blood protein levels and pulse pressure (SNP rs7577293, P = 5 × 10^−76^ and rs11689667, P = 2 × 10^−08^, respectively; Supplementary Table 9).

### Genetic risk score

To date, 99 variants could be robustly identified to predispose for CAD according the current literature, (Supplementary Table 10). For all variants except SNP rs1878406, we had genotypes available in both case and control cohorts in the AgP-Ger sample and no genotypes were missing. Thus, the CAD-GRS, based on 98 CAD risk variants, was calculated in the German cases-control sample for AgP and revealed no differences in mean GRS between AgP-Ger cases (μ = 107.46; σ^2^ = 6.43) and controls (μ = 107.48; σ^2^ = 6.48; Supplementary Fig. 1).

## Discussion

In this study, we aimed to identify novel genetic risk factors that are shared between CAD and PD, in order to improve the current pathogenic understanding of both diseases and highlight possible common biological underpinnings. We provide evidence for an association with PD for SNP rs1561198, located at *VAMP8*, which is a known genetic risk variant of CAD. We were able to replicate our observation from the discovery sample in an independent case-control sample of PD.

A limitation of the study was the relatively small sizes of the available PD case-controls samples compared to the CARDIoGRAMplusC4D sample. The CARDIoGRAMplusC4D sample was ~12 times larger than the pooled PD samples. This is why we consider that the association of SNP rs1561198 reached genome-wide significance in CAD, whereas the same variant with a similar effect size had a significance level in the pooled PD sample several orders of magnitudes lower. However, despite the comparatively lower association P-value of rs1561198 with PD with P = 2.02 × 10^−4^ in the pooled PD samples, the association passed the Bonferroni-corrected significance threshold in the replication and we conclude that the association of rs1561198 with PD is a true positive finding. Additional evidence for association is provided by the observation that rs1561198 – together with several highly linked variants - showed consistent association of the same risk allele with PD in all individual PD samples, with the exception of the Dutch AgP sample that included the smallest number of cases (n = 179), but a comparably large number of controls (n = 2,891). Because of the low number of cases, we argue that this sample is highly susceptible to random errors (i.e. chance effects) and, at the same time, results in effect confidence intervals for which the sizes are biased by the large control sample. For the same reason we think, that the inter-sample heterogeneity would have increased when adding the Dutch AgP case-control sample to the discovery stage and deterred us from using the fixed effects model in this stage. Because of that and to decrease the chances of false positive association findings transferred from the discovery stage to the replication, we decided to exclude the Dutch sample from the discovery stage, but to add it to the replication stage instead.

In the discovery stage, the LD block of rs1561198 had an OR of ~1.19 in AgP-Ger, which is close to the estimations of detectable ORs according to our power calculations. Compared with the CAD (OR = 1.06) and CP samples (OR = 1.09) that were included in our analyses, the higher OR in AgP-Ger might be exclusive to AgP, which is influenced to greater extent by genetic factors compared to the late onset diseases CAD and CP, or reflect the Winner’s curse bias, which results in an overestimation of the effect size.

The power of our study is also demonstrated by the re-discovery of the known association at 9p21.3 (*CDKN2B-AS1*), which is well established for both CAD^4–6^ and PD^7^. The lead-SNP of the association of this locus, rs10116277, had an OR = 1.26 (MAF = 52%) in AgP-Ger, which was comparable in size and direction to the CAD association (OR = 1.21). This SNP is among the variants with the highest OR of all currently known genetic loci of CAD and AgP (Supplementary Table 9)^49^. The other previously reported shared risk loci at *VAMP3* (rs10864294)^10^ and *PLG* (rs4252120)^9^ were not re-discovered because they did not pass the pre-defined criterion P_CAD_ < 5 × 10^−8^.

Our finding of shared risk variants at *VAMP8* support and complement results of a previous report addressing the shared molecular mechanisms of both diseases. In this study, a transcriptome-wide shRNA knock-down approach demonstrated that *CDKN2B-AS1* and *VAMP3* expression is correlated on the RNA and protein level, and a rare variant upstream of *VAMP3* (rs17030881) was suggested to be associated with AgP and CAD^10^. *VAMP3* and *VAMP8* are simultaneously expressed in various cell types, e.g. in the secretory granules of platelets^50^ and mast cells^51^, where they form complexes with platelet syntaxin 4 during platelet secretion, to release inflammatory cytokines, or in adipocytes, where they promote the fusion of glucose transporter type 4 (*GLUT4*)^52^. *VAMP3* and *VAMP8* mediated membrane trafficking in platelets also plays an important role in thrombosis and wound healing, processes with established relevance for the etiology of CAD and PD. In addition, VAMP mediated platelet secretion has an antimicrobial role in the host response to bacterial infection (previously reviewed^53,54^).

PD is caused by bacterial pathogens and it was suggested that the risk of CAD is putatively increased by bacterial infections^55,56^. Likewise, two clinical trials involving full-mouth mechanical debridement resulted in a transient deterioration of surrogate markers of CAD^57,58^, which supports the notion that bacterial inoculation and other procedure-related inflammation resulting from mechanical debridement has immediate negative effects on risk for CAD. In addition, living oral pathogens were detected in atheromatous plaques from coronary arteries^59,60^. In this context it is interesting that various studies determined that SNARE (soluble N-ethylmaleimide-sensitive factor attachment receptor) proteins like VAMP3 and VAMP8, are manipulated by specific pathogens to corrupt host membrane vesicular trafficking (reviewed in^61^). E.g., *Chlamydia* species mimic VAMP3 and VAMP8 to establish within the host cell^62^, *Shigella* species stimulate endocytosis of VAMP3 and VAMP8 in the internalization process of Shiga toxin^63^, and virulence factors of *Leishmania* parasites cleave VAMP3 and VAMP8 to evade phagocytosis^64^. We also note that a GWAS on periodontal pathogen colonization reported a region within *CAMTA1*, adjacent to *VAMP3*, to be associated with increased quantities of oral pathogenic bacteria^11,65^. Taken together, we note a possible yet speculative link of the observed associations with *VAMP3* and *VAMP8* and the increased risk of PD and CAD with effects of the putative causative variants on functions of coagulation, wound healing and thrombosis, as well as the invasion of some bacteria into host cells. However, the putative causal variant(s) of the *VAMP8* associated haplotype block and their likely effects on gene regulation still need to be identified. Currently, their effects in the susceptibility for CAD and PD are speculative.

We conclude that our finding of a haplotype block at *VAMP8*, which increases the risk for CAD and PD adds to the previously reported shared risk variants and indicates that the observed association of CAD and PD reported in prior epidemiological studies (previously reviewed^2^) cannot be solely explained by shared environmental risk factors. In addition, the current study probably missed possible associations with a shared role in the genetic etiology of CAD and PD. This is because of a lack of statistical power of PD to identify shared risk variant of CAD and PD with ORs <1.1 and an incomplete coverage of common and rare variants in the GWAS data sets. To identify the full spectrum of pleiotropic variants, larger sample sizes are needed for future studies.

## Electronic supplementary material

Supplementary Material

Supplementary Material (Excel Sheet)

## Data Availability

The CARDIoGRAMplusC4D summary statistics dataset is freely available on the consortium website (http://www.cardiogramplusc4d.org). Similarly, summary statistics for CP-EU-mod and CP-EU-sev can also be downloaded (see respective publication). Genotype data for aggressive periodontitis samples and the chronic periodontitis sample with German descent are available upon request from the biobanks PopGen (https://www.epidemiologie.uni-kiel.de/biobanking) and ShIP (https://www.fvcm.med.uni-greifswald.de/index.html).
