# Supplementary material for: Genome-wide association meta-analysis of coronary artery disease and periodontitis reveals a novel shared risk locus
Source: Sci Rep. 2018 Sep 12;8:13678. doi: 10.1038/s41598-018-31980-8 (PMC6135769; doi:10.1038/s41598-018-31980-8)

# Supplementary Material: Genome-wide association meta-analysis of coronary artery disease and periodontitis reveals a novel shared risk locus

Matthias Munz^1,2,3^, Gesa M. Richter^1^, Bruno G. Loos^4^, Søren Jepsen^5^, Kimon Divaris^6,7^, Steven Offenbacher^8^, Alexander Teumer^9^, Birte Holtfreter^10^, Thomas Kocher^10^, Corinna Bruckmann^11^, Yvonne Jockel-Schneider^12^, Christian Graetz^13^, Loreto Munoz^2,3,14^ , Anita Bhandari^2,3,14^, Stephanie Tennstedt^2,3,14^, Ingmar Staufenbiel^15^, Nathalie van der Velde^16,17^, André G. Uitterlinden^16^, Lisette C.P.G.M. de Groot^18^, Jürgen Wellmann^19^, Klaus Berger^19^, Bastian Krone^20^, Per Hoffmann^21,22^, Matthias Laudes^23^, Wolfgang Lieb^24^, Andre Franke^24^, Henrik Dommisch^1^, Jeanette Erdmann^2,3,14,¶^, Arne S. Schaefer^1,¶,*^

^¶^these authors contributed equally

**Affiliations:**

^1^Charité – University Medicine Berlin, corporate member of Freie Universität Berlin, Humboldt-Universität zu Berlin, and Berlin Institute of Health, Institute for Dental and Craniofacial Sciences, Department of Periodontology and Synoptic Dentistry, Berlin, Germany
^2^Institute for Cardiogenetics, University of Lübeck, 23562 Lübeck, Germany
^3^DZHK (German Research Centre for Cardiovascular Research), partner site Hamburg/Lübeck/Kiel, 23562 Lübeck, Germany
^4^Department of Periodontology and Oral Biochemistry, Academic Centre for Dentistry Amsterdam (ACTA), University of Amsterdam and Vrije Universiteit Amsterdam, The Netherlands
^5^Department of Periodontology, Operative and Preventive Dentistry, University of Bonn, Bonn, Germany
^6^Department of Pediatric Dentistry, School of Dentistry, University of North Carolina at Chapel Hill, Chapel Hill, USA
^7^Department of Epidemiology, Gillings School of Global Public Health, University of North Carolina at Chapel Hill, Chapel Hill, USA
^8^Department of Periodontology, School of Dentistry, University of North Carolina at Chapel Hill, Chapel Hill, USA
^9^Institute for Community Medicine, University Medicine Greifswald, Greifswald, Germany
^10^Unit of Periodontology, Department of Restorative Dentistry, Periodontology, Endodontology, Preventive Dentistry and Pedodontics, Dental School, University Medicine Greifswald, Germany
^11^Department of Conservative Dentistry and Periodontology, Medical University Vienna, School of Dentistry, Vienna, Austria
^12^Department of Periodontology, Clinic of Preventive Dentistry and Periodontology, University Medical Center of the Julius-Maximilians-University, Würzburg, Germany
^13^Department of Conservative Dentistry, Unit of Periodontology, University Medical Center Schleswig-Holstein, Campus Kiel, Germany
^14^University Heart Center Luebeck, 23562 Lübeck, Germany
^15^Department of Conservative Dentistry, Periodontology and Preventive Dentistry, Hannover Medical School, Hannover, Germany
^16^Department of Internal Medicine, Erasmus Medical Center, Rotterdam, The Netherlands
^17^Department of Internal Medicine section of Geriatrics, Amsterdam Medical Center, Amsterdam, The Netherlands
^18^Wageningen University, Division of Human Nutrition, Wageningen, the Netherlands
^19^Institute of Epidemiology and Social Medicine, University Münster, Germany
^20^Institute of Medical Informatics, Biometry and Epidemiology, University Clinic Essen, Germany
^21^Institute of Human Genetics, University of Bonn, Germany
^22^Human Genomics Research Group, Department of Biomedicine, University Hospital of Basel, Switzerland
^23^Department of Medicine 1, University of Kiel, Germany
^24^Institute of Epidemiology, Christian-Albrechts-University, Kiel, Germany

^*^Corresponding author

Email: arne.schaefer@charite.de
Telephone: +49 30 450 562 343
Fax: +49 30 450 7562 343

Address:
Charité – Universitätsmedizin Berlin, corporate member of Freie Universität Berlin, Humboldt-Universität zu Berlin, and Berlin Institute of Health, Institute for Dental and Craniofacial Sciences, Department of Periodontology and Synoptic Dentistry, Berlin, Germany
Aßmannshauser Straße 4-6
14197 Berlin, Germany

**Supplementary Table 1.** 220 variants in three distinct loci passed our criteria in the discovery stage. See Excel sheet.

**Supplementary Table 2.** According to 1000 Genomes Phase 3 EUR data, the lead SNPs rs4468572 and rs1561198 at 15q25.1 and 2p11.2 have altogether 75 variants in high LD (r^2^ > 0.8). See Excel sheet.

**Supplementary Table 3.** Replication of the variants at 15q25.1 and 2p11.2 that were identified in the discovery stage. See Excel sheet.

**Supplementary Table 4.** Meta-analysis of CP-EU-mod and CP-EU-sev to compare the inflation of P-value with and without adjustment for shared controls.

**Supplementary Table 5.** Genes that are sharing TADs with the high LD block of discovery lead SNP rs1561198 at 2p11.2. See Excel sheet.

**Supplementary Table 6.** Public eQTL data indicated c*is*- and *trans*- regulatory effects on several genes for the high LD block of discovery lead SNP rs1561198 at 2p11.2. See Excel sheet.

**Supplementary Table 7.** Consequence types and CADD scores of the variants in the high LD block of discovery lead SNP rs1561198 at 2p11.2. See Excel sheet.

**Supplementary Table 8.** Associations in the NHGRI-EBI GWAS Catalog for variants in the high LD block of discovery lead SNP rs1561198. See Excel sheet.

**Supplementary Table 9.** Associations in the NHGRI-EBI GWAS Catalog at discovery lead SNP rs1561198 +/- 500 kb. See Excel sheet.

**Supplementary Table 10.** List of 99 CAD risk variants. See Excel sheet.

**Supplementary Figure 1.** Distribution of the GRS scores for cases and controls in the AgP-Ger sample.


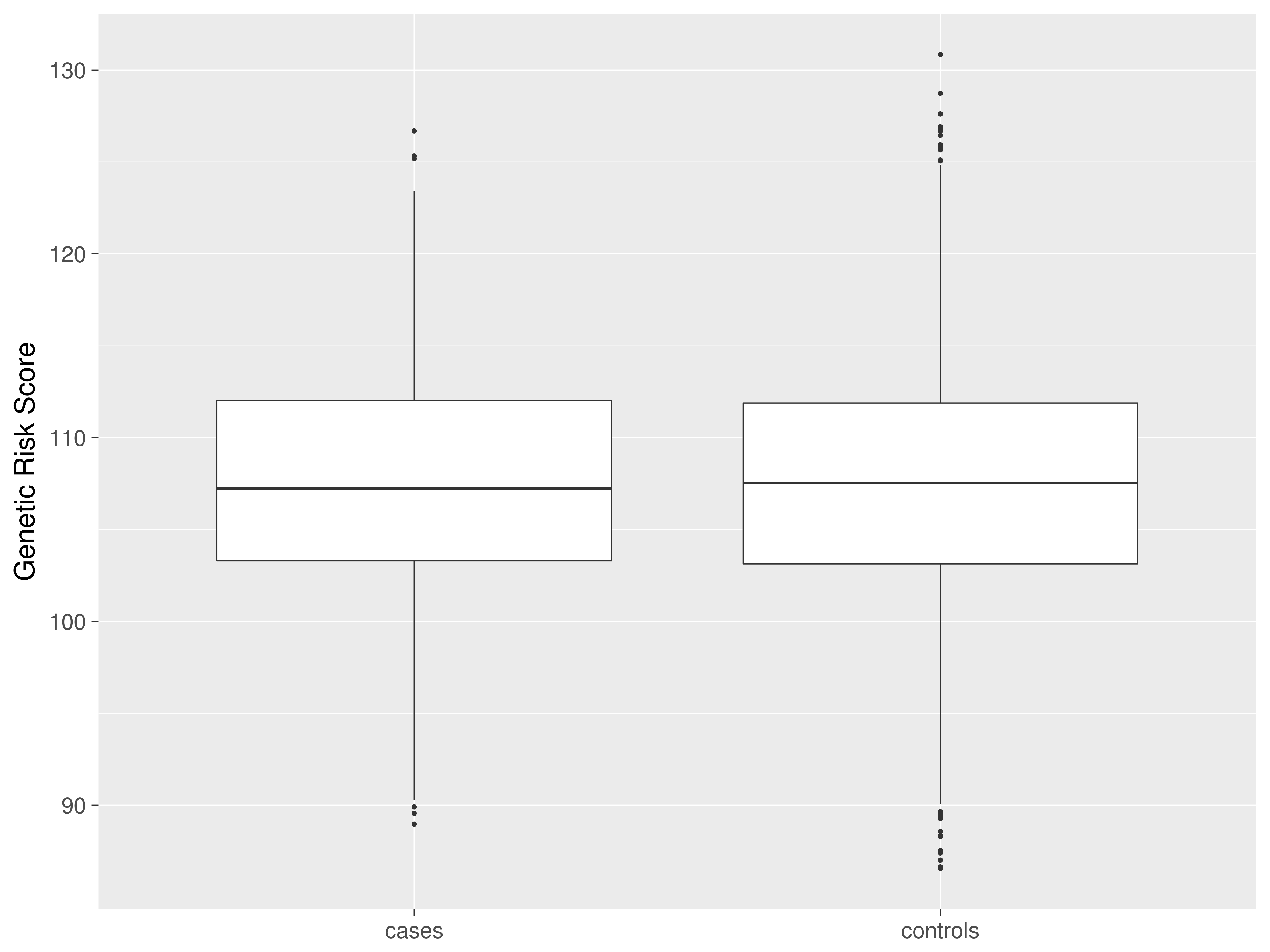

Supplement: Supplementary file 1 — Supplementary Material [file 41598_2018_31980_MOESM1_ESM.doc]
